# Salivary Protein 1 of Brown Planthopper Is Required for Survival and Induces Immunity Response in Plants

**DOI:** 10.3389/fpls.2020.571280

**Published:** 2020-08-27

**Authors:** Jin Huang, Ning Zhang, Junhan Shan, Yaxin Peng, Jianping Guo, Cong Zhou, Shaojie Shi, Xiaohong Zheng, Di Wu, Wei Guan, Ke Yang, Bo Du, Lili Zhu, Longping Yuan, Guangcun He, Rongzhi Chen

**Affiliations:** ^1^State Key Laboratory of Hybrid Rice, College of Life Sciences, Wuhan University, Wuhan, China; ^2^State Key Laboratory of Hybrid Rice, Hunan Hybrid Rice Research Center, Hunan Academy of Agricultural Sciences, Changsha, China

**Keywords:** brown planthopper, salivary proteins, RNA interference, insect-plant interaction, plant defense responses

## Abstract

The brown planthopper (BPH), *Nilaparvata lugens* Stål, is one of the major pests of rice. It uses its stylet to penetrate rice phloem, feeding on rice sap and causing direct damage to rice or even plant death. During the feeding process, BPHs secrete saliva into plant tissues, which plays crucial roles in the plant-insect interactions. However, little is known about how the salivary proteins secreted by BPH affect feeding ability and how they induce plant immune responses. Here, we identified an *N. lugens* Salivary Protein 1 (NlSP1) by screening salivary proteome and characterized its functions in BPH and plants. NlSP1 induces cell death, H_2_O_2_ accumulation, the expression of defense-related genes, and callose deposition *in planta*. The active region of NlSP1 that induces plant cell death is located in its N-terminal region. Inhibition of *NlSP1* expression in BPHs reduced their feeding ability and had a lethal effect on them. Most importantly, we demonstrated that NlSP1 was able to be secreted into rice plant during feeding process and form a complex with certain interacting partner of rice. These results provide a detailed characterization of a salivary protein from BPHs and offers new insights into our understanding of rice-BPH interaction.

## Introduction

The war between plants and herbivorous insects has a long history and continues. Herbivorous insects prey on plants by chewing or piercing-sucking and plants defense against herbivorous insects (Ehrlich and Raven, 1964). To protect themselves from injury by herbivores, plants have evolved sophisticated systems for resistance to herbivorous insects, including constitutive and induced defenses (Felton and Tumlinson, 2008; Erb et al., 2012; Mithöefer and Boland, 2012; Stam et al., 2014; Schuman and Baldwin, 2016). Constitutive defenses are the physical and chemical defense characteristic of plants without the influence of herbivorous insects. In contrast, induced defenses are installed only after the plant attacked by herbivores (Wu and Baldwin, 2010). Inducible defenses are mainly initiated by the recognition of saliva or oral secretions of herbivores and are followed by the activation of a complex signaling network, including reactive oxygen species (ROS) production, calcium signaling, mitogen-activated protein kinase (MAPK) cascades, and JA, SA, ethylene, and hypersensitive response (HR) pathways (Erb and Reymond, 2019; Wilkinson et al., 2019).

As with plant-pathogen interactions, herbivore-associated molecular patterns (HAMPs) and effectors work on recognition of plant and insect and activation of plant defense responses (Wu and Baldwin, 2010; Hogenhout and Bos, 2011; Jiang et al., 2019). To date, many HAMPs have been identified in saliva, regurgitant, and egg secretions of herbivores, including fatty acid conjugates, caeliferins, bruchins, inceptins, and salivary enzymes, such as β-glucosidase and lipase (Wu and Baldwin, 2010; Erb et al., 2012; Acevedo et al., 2015). In addition, the mechanism of HAMPs inducing the defense response has also been extensively studied (Schäfer et al., 2011; Erb et al., 2012; Stahl et al., 2018; Wari et al., 2019). By contrast, there have been fewer studies on herbivores effectors. Broadly speaking, small molecules of all pathogen or insect proteins secreted into host cells that alter host structure and function are defined as effectors (Hogenhout et al., 2009). It has been reported that glucose oxidase (GOX) in *Helicoverpa zea* salivary glands suppressed host defenses, which inhibited nicotine production in tobacco (*Nicotiana tabacum*) and generated H_2_O_2_ from D-glucose (Musser et al., 2002; Diezel et al., 2009). Moreover, expression of the aphid effectors, Mp10 and Mp42, in host plants decreases the fecundity of green peach aphid *Acyrthosiphon pisum*, while other effectors, C002 and Mp55, enhances aphid fecundity (Bos et al., 2010; Pitino et al., 2011; Elzinga et al., 2014). Similarly, other aphid effectors, such as calcium-binding proteins from the vetch aphid (*Megoura viciae*; Will et al., 2007), structural sheath proteins from the grain aphid (*Sitobion avenae*; Abdellatef et al., 2015), Me10 and Me23 from the potato aphid (*Macrosiphum euphorbiae*; Atamian et al., 2013), Armet from the pea aphid (*Acyrthosiphon pisum*; Wang et al., 2015), and MpMIF from the pea aphid (Naessens et al., 2015), also have been shown to improve aphid performance. However, the functions of effectors from piercing-sucking herbivores other than aphids remains poorly understood.

The brown planthopper (BPH), *Nilaparvata lugens* Stål, is a kind of typical phloem sap-sucking insects. As one of the most devastating insect pests in rice-growing countries and regions in Asia, BPH causes heavy yield losses and economic damage to rice by directly feeding on rice or indirectly transmitting viral diseases (Settle et al., 1996; Jena and Kim, 2010). BPHs puncture into the tissue of rice plants *via* using their stylets and then go deep into the phloem to suck the sap for nutrients (Wang et al., 2008). During this feeding process, BPHs repeatedly secrete saliva to make them easier to feed. As with other piercing-sucking insects, BPH secretes two primary kinds of saliva during feeding: watery and gelling (Sogawa et al., 1982). Watery saliva contains various detoxification enzymes, proteases, and proteins that interact with plants (HAMPs and effectors; Konishi et al., 2009). Gelling saliva quickly solidifies following secretion and forms a continuous salivary sheath around BPHs’ stylets, providing support and lubrication for the stylets (Wang et al., 2008). Thus, the saliva of BPH plays a crucial role their interaction with the host plants (Miles, 1999; Ye et al., 2017). Up to present, proteomics of BPH saliva and transcriptomics of BPH salivary glands have been identified and analyzed (Noda et al., 2008; Konishi et al., 2009; Ji et al., 2013; Huang et al., 2016; Liu et al., 2016). Several salivary proteins, such as NlShp, NlEG1, NlSEF1, and NlMLP, have been found to play a role in salivary sheath formation and/or BPH feeding (Huang et al., 2015; Ji et al., 2017; Ye et al., 2017; Shangguan et al., 2018). Other salivary proteins, such as Nl12, Nl16, Nl28, and Nl43, have been shown to activate plant defensive responses (Rao et al., 2019). However, increased efforts are needed to make major advances in this important and historically understudied area of research (Jiang et al., 2019).

By proteome analysis and *in planta* functional assays of BPH secreted salivary proteins, we identified a secreted Salivary Protein 1 (NlSP1). NlSP1 is necessary for the survival of BPH and plays a role in BPH feeding. NlSP1 induces various defense responses in plants, including cell death, ROS generation, the expression of defense-related genes, and callose deposition. The functional motif is located in the amino terminus of NlSP1. Importantly, NlSP1 can be secreted into rice plant during feeding and form a complex with certain interacting partner of rice. Our results provide new insights into the understanding of rice-BPH interactions at the molecular level.

## Materials and Methods

### Insects and Plants

The BPH insect populations were reared on rice seedlings of the susceptible cultivar TaichungNative 1 (TN1) in the laboratory under controlled environmental conditions (26°C ± 1°C, 16-h-light/8-h-dark photoperiod) at Wuhan University, China. Tobacco (*Nicotiana benthamiana*) plants were grown in growth chambers under long day (16 h light) conditions at 25°C with 60% to 75% relative humidity. The japonica rice (*Oryza sativa*) variety Nipponbare was grown in the experimental fields at Wuhan University Institute of Genetics and was used as the transgenic acceptor and as a susceptible rice control.

### Collection and Concentration of Salivary Protein and LC−MS/MS Analysis

Two membranes of stretched Parafilm M Laboratory Films (Neenah, USA) that contained 500 µL of 2.5% sucrose in Milli-Q water were attached to a cylindrical PVC pipe (2 cm × 5 cm). Twenty third-instar BPH nymphs were transferred from rice seedlings into each PVC pipe for 24 h at 28°C. About 4000 nymphs were used for each biological repeat. The liquid containing watery saliva from the space between the two layers of Parafilm Film was collected with a pipet after feeding on dietary sucrose. The salivary sheaths remaining on the membrane after BPH feeding were carefully collected by scrapping off them with a small spoon in 500 µL Milli-Q water each device. The collected dilute salivary protein solutions were concentrated by vacuum drying method and chloroform/methanol method. Concentrated protein samples of watery and gelling saliva were separated by SDS-PAGE gel electrophoresis with a 5% stacking and 12% separating gel (Sigma-Aldrich, USA), and stained with 0.025% Coomassie Brilliant blue R-250 (Sigma-Aldrich, USA). The mixed protein sample was digested with trypsin in 50 mM NH_4_HCO_3_ buffer overnight at 37°C. A LTQ VELOS mass spectrometer (Thermo Finnigan, San Jose, CA) was used for liquid chromatography-tandem mass spectrometry (LC-MS/MS) at center for proteomics research and analysis of Shanghai Applied Protein Technology Co, Ltd. Protein identification was performed using MASCOT software (version 2.2, Matrix Science, Boston, USA) against the transcriptomic database of *N. lugens* salivary glands (containing 18,099 protein-coding sequences; Rao et al., 2019) and another transcriptomic database of whole body and salivary glands of *N. lugens* (NLWB, 16,440 predicted protein sequences; NLSG, 14,203 predicted protein sequences; Liu et al., 2016).

### Cloning of Candidate BPH Effectors and Plasmid Construction

Routine molecular cloning techniques were used to prepare the constructs. The primers used in this work are listed in **Supplemental Table S3**.

All of the resulting recombinant vectors were sequenced. According to the cDNA sequences in the transcriptome of salivary proteins, the corresponding ORF primers of the candidate salivary proteins were designed, and the ORFs were amplified by PCR from cDNA of BPH biotype I. The PCR products were ligated into the pMD18-T vector to obtain the accurate ORF sequences of the candidate protein by sequencing. Some of the candidate salivary proteins have no intact cDNA sequences in the transcriptome of salivary proteins. We obtain the full-length cDNA of the candidate salivary proteins by using 5′-Full RACE Kit and 3′-Full RACE Core Set (Takara, China) according to the manufacturer’s instructions.

For construction of the Gateway entry clones, the ORFs of candidate BPH effector were amplified with primers flanked by two attB sites and transferred into pDONR207 by BP Clonase II enzyme mix (Invitrogen). The entry vectors were recombined into the destination vector pEarleyGate100 by LR Clonase II enzyme mix (Invitrogen). The resulting expression constructs were used for cell death assays in *N. benthamiana*. Similarly, NlSP1 and its derived deletion mutants were recombined into the destination vector pEarleyGate101 (with a C-terminal YFP-HA epitope tag). The constructs of pEarleyGate101 vector contained GFP or NlMLP are available in previously published article (Shangguan et al., 2018). The resulting pEarleyGate101 constructs were used for rice protoplast transformation and *N. benthamiana* agroinfiltration experiments.

For protein expression and purification in the preparation of polyclonal antibodies, the coding sequence of NlSP1 without the predicted signal peptide was cloned into the BamHI and sites EcoRI of pET-28a (EMD Biosciences, Novagen), yielding constructs designated 6×His-NlSP1.

For protein expression in yeast, the destination vector pGBKT7-GW is constructed by adding Gateway system element to the *NdeI* and *BamHI* sites of pGBKT7. The entry vector containing the coding sequence of NlSP1 without the predicted signal peptide was recombined into the destination vector pGBKT7-GW by LR Clonase II enzyme mix (Invitrogen). The resulting construct was used for yeast transformation and expression.

For subcellular localization in rice protoplasts, the coding sequence of NlSP1 without the predicted signal peptide was cloned into the Gateway intermediate vector pDONR207 *via* BP reaction, and then recombined into plant expression vector pGWB554 (Nakagawa et al., 2007) by LR reaction to be under the control of the 35S promoter and fused to a C-terminal mRFP tag. The resulting construct designated NlSP1-RFP. Nucleus marker was bZIP63-GFP (constructed by this experiment) as described previously (Walter et al., 2004). Moreover, other organelle makers were peroxisome marker (CD3-979, FP-PTS1), ER marker (CD3-955, AtWAK2-HDEL), GA marker (CD3-963, Man49), mitochondrial marker (CD3-987, ScCOX4), and tonoplast marker (CD3-971, γ-TIP) as described previously (Nelson et al., 2007).

For constitutively express *NlSP1-dsRNA* in rice, a 500-bp template fragment and a PDK intron were used to generate a hairpin RNAi construct as described previously (Zha et al., 2011). The construct was cloned into plant expression vector pCXUN (accession no. FJ905215) under the control of the plant ubiquitin promoter.

### RNA Isolation and qRT-PCR

Total RNA was extracted from the following materials: (1) different BPH tissue samples (salivary glands, midguts, fat bodies, and the remaining parts) that had been dissected from BPH female adults using a stereomicroscope; (2) whole bodies of BPH at different developmental stages, including from first to fifth instar nymphs, female adults, and male adults. Total RNA was isolated using the RNAiso Plus kit (TaKaRa) according to manufacturer’s instructions. All RNA samples were reverse-transcribed into cDNAs using the PrimeScript RT Reagent Kit with gDNA Eraser (Takara). The qRT-PCR assays were performed on the Bio-Rad CFX-96 Real-Time PCR system with the iTaq Universal SYBR Green Supermix Kit (Bio-Rad). The BPH housekeeping gene β-actin was used as an internal standard to normalize cDNA concentrations. Relative expression ratios were calculated using the Pfaffl method (Pfaffl, 2001). The primers used for target genes expression analysis are listed in **Supplementary Table S4**. Three independent biological replicates were analyzed in each experiment.

### Expression of NlSP1 in *Escherichia coli* and Anti-NlSP1 Polyclonal Antibody Production

The recombinant vector NlSP1:pET-28a was transformed into *E. coli* BL21 (DE3) strain. Expression of recombinant protein was induced by adding IPTG (0.1 mM final concentration) at 16°C. The protein product was purified by using Ni-NTA columns (CWbio, China) according to the manufacturer’s instructions. The purified products were concentrated with a Amicon^®^ Ultra-15 centrifugal filter device (Millipore, USA) to remove imidazole. The final purified concentrated products mixed with 5× SDS loading buffer, separated by SDS-PAGE in a 10% acrylamide gel (sigma-aldrich, USA), and stained with 0.025% Coomassie Brilliant blue R-250 (sigma-aldrich, USA) in water. The induced protein of NlSP1 was validated by Western blotting with anti-HIS (Roche, Switzerland). The purified protein of NlSP1 was selected as the antigen, and the polyclonal rabbit antibodies of NlSP1 were made and purified by Dia-An Biotech, Inc, in Wuhan, China. Western blotting was conducted to verify the antibody specificity.

### Protein Extraction and Immunoblot Analysis

Proteins from BPHs were extracted from the whole bodies and homogenized in 100 *μ*L of SDS protein extraction buffer (10 mM Tris-HCl pH 8.0, 5 mM EDTA, 10% SDS, with 10 mM DTT and 1mM PMSF added immediately before use). After incubation on ice for 1 h, the homogenate was centrifuged at 4°C at 20,000*g* for 15 min. Ten *μ*L of the resulting supernatant were separated by 10% SDS-PAGE gels. Immunoblotting was performed with anti-NlSP1 polyclonal antibody described above at a dilution ratio of 1:1000 with dilution buffer (20 mM Tris-HCl, pH 7.4, 150 mM NaCl, 0.1% Tween 20, and 3% BSA) followed by horseradish peroxidase (HRP)-conjugated goat anti-rabbit antibodies (1:7,500 dilution). The HRP signals were detected using an Immobilon western chemiluminescent HRP substrate kit (Millpore).

Proteins from rice plants were extracted from the leaf sheaths of 25-d-old seedling for immunoblotting. 0.2 g samples were ground to powder with liquid nitrogen and homogenized in 300 *μ*L of rice protein extraction buffer (100 mM Tris-HCl pH 7.5, 1mM EDTA, 5 mM MgCl_2_, and 0.5% Triton X-100, with 2 mM DTT and 1 mM PMSF added immediately before use). After incubation on ice for 2 h, the homogenate was centrifuged at 4°C at 20,000g for 15 min, and the resulting supernatant was used for immunoblot analysis performed as previously described (Hu et al., 2011). Protein expression was detected by immunoblotting using anti-NlSP1 polyclonal antibody at a dilution ratio of 1:100 with dilution buffer described above followed by HRP-conjugated goat anti-rabbit antibodies (1:7,500 dilution). The HRP signals were detected using an Immobilon western chemiluminescent HRP substrate kit (Millpore).

Protein extracts from rice protoplasts were prepared as described below. Protoplast samples were collected 16 h following transformation from three tubes of protoplasts. Each sample was extracted in 200 *μ*L rice protein extraction buffer described above and homogenized by vortex. After incubation on ice for 1 h, the homogenate was centrifuged at 4°C at 20,000*g* for 15 min. Ten *μ*L of the resulting supernatant were separated by 10% SDS-PAGE gels. Immunoblotting was performed with anti-HA antibody (MBL, Japan) at a dilution ratio of 1:1,000 with dilution buffer described above followed by HRP-conjugated goat anti-mouse antibodies (1:7,500 dilution). The HRP signals were detected using an Immobilon western chemiluminescent HRP substrate kit (Millpore).

Proteins extracts from *N. benthamiana* leaves were prepared as described below. Leaf samples were collected 48 h after agroinfiltration. Three 10-mm-diameter leaf discs taken from the infiltrated areas were homogenized by pestles with 100 *μ*L SDS protein extraction buffer described above. After boiled for 10 min, the homogenate was centrifuged at 4°C at 20,000*g* for 15 min. Ten *μ*L of the resulting supernatant were separated by 10% SDS-PAGE gels. Immunoblotting was performed with anti-HA antibody (MBL, Japan) at a dilution ratio of 1:1,000 with dilution buffer described above followed by HRP-conjugated goat anti-mouse antibodies (1:7,500 dilution). The HRP signals were detected using an Immobilon western chemiluminescent HRP substrate kit (Millpore).

### Cell Death Assays and Subcellular Localization in Rice Protoplasts

Transient expression in rice protoplasts was implemented as previous described (Zhang et al., 2011). The cell death assays were conducted using rice protoplasts as described (Zhao et al., 2016). For the cell viability assay, protoplasts were transfected with the indicated plasmids for 20 h and stained with 220 *μ*g/mL fluorescein diacetate (FDA). Each protoplast sample was scored under fluorescence microscope (TCS SP8, Lecia) in at least 10 randomly selected microscopic fields. For the luciferase assay, The Renilla luciferase gene was used as a reporter to monitor protoplast viability. The indicated genes and LUC gene were co-transformed in rice protoplasts with the same quantity level of cells, respectively. Luciferase activity was measured 40 h following transformation using a Renilla luciferase assay system (Promega).

For subcellular localization, Protoplasts preparation were the same as described above. NlSP1-RFP recombinant vector was co-transformed with nuclear-GFP maker bZIP63 (Walter et al., 2004) and others organelles makers (Nelson et al., 2007) in rice protoplasts, respectively. After transfection, the protoplasts were placed in a 28°C dark incubator and cultured for 16 to 22 h. The fluorescence of protoplasts was observed and photographed using Leica TCS SP8 confocal fluorescence microscope.

### *Agrobacterium*-Mediated Infiltration Assays of *N. benthamiana* Leaves

Constructs were introduced into *Agrobacterium tumefaciens* strain GV3101 *via* electroporation. The recombinant strains were cultured in Luria-Bertani (LB) medium supplemented with appropriate antibiotics for 24 h at 28°C with shaking at 200 rpm. The cells were harvested by centrifugation at 5,000g for 5 min and resuspended in infiltration medium (10 mM MES, pH 5.7, 10 mM MgCl_2_, and 150 mM acetosyringone). The suspension was adjusted OD_600_ to 0.2 for cell death assay and 0.5 for other experiments, and cultured in the dark at 28°C for 1 to 3 h. Finally, the suspension was infiltrated into 4-week-old N. benthamiana leaves using a needleless syringes for expression.

Cell death symptom development in *N. benthamiana* leaves was observed visually and photographed 3 to 5 d after infiltration. Cell death was also assayed by measuring ion leakage and trypan blue staining. For ion leakage assay, four leaf discs (10 mm diameter) of *N. benthamiana* leaves 48 h after infiltration were placed into 5 mL of ultrapure water. Each sample was incubated at room temperature overnight, and its ion conductivity EC1 of the bathing solution was measured using a conductivity meter (FiveGO-FG3). After boiled 10 min, its conductivity EC2 was measured after cooling to room temperature. Relative electrolyte leakage (%) = 100 × EC1/EC2. The experiments were implemented four times. For trypan blue staining assay, *N. benthamiana* leaves after infiltration were placed in staining solution (10 mL lactic acid, 10 mL glycerol, 10 g phenol, 10 mL H_2_O, 15 mg trypan blue) mixed with the same volume of absolute ethyl alcohol. After vacuum filtration, the leaves were boiled for 10 min and cooled to room temperature for overnight. Then, the samples were decolorized in 2.5 g/mL chloral hydrate to remove the background and photographed.

ROS levels were measured according to H_2_O_2_ accumulation after staining *N. benthamiana* leaves with DAB. Agroinfiltrated *N. benthamiana* leaves were placed in DAB staining solution (1 mg/mL DAB) and maintained for overnight at 25°C. Then the leaf tissues were boiled in 95% ethanol for 15 min until all the tissue was entirely bleached. The blenched samples were then immersed in absolute ethyl alcohol to further clear the background. The experiment was performed three times.

Callose deposition in leaf discs 48 h after infiltration was visualized by Aniline Blue staining as previously described (Naessens et al., 2015). In short, the discated discs were soaked in ethanol in order of 50%, 70%, 95% and 100%, and bleached continuously for 2 h per wash. Then the bleached samples were soaked in 70% ethanol, 50% ethanol and distilled-deionized water successively for rehydration. Callose of the rehydrated samples was stained by incubated in Aniline Blue solution (70 mM KH_2_PO_4_ and 0.05% Aniline Blue, pH 9) for 1 h. Stained leaf discs were mounted in 80% glycerol and observed with a Laser Scanning Confocal Microscope (TCS SP8, Lecia). The number of callose deposits was counted using ImageJ software.

### RNAi Experiments and BPH Bioassays

A 500-bp fragment of *NlSP1* and a 657-bp fragment of control gene *GFP* were amplified by PCR with primers including a T7 promoter sequence (list of primers in **Supplemental Table S3**). The PCR products were synthesized using the MEGAscript T7 High Yield Transcription Kit (Ambion, USA) according to the manufacturer’s instructions. The dsRNAs were purified by phenol chloroform extraction and concentrated by sodium acetate solution, then resuspended in nuclease-free water at a concentration of 5 mg/mL. Third- or fifth-instar nymphs were injected at the conjunction of the prothorax and mesothorax using a microprocessor-controlled Nanoliter 2010 injector (World Precision Instruments), under a stereoscopic microscope (Olmpus). A 46-nL volume of dsRNA of *NlSP1* or *GFP*, or nuclease-free water was injected into each nymph. To determine the efficiency of gene silencing after dsRNA injection, *NlSP1* transcription and NlSP1 protein expression of BPHs after injection with dsRNA of *NlSP1* or *GFP*, or nuclease-free water were measured at 1 to 5 days after injection, respectively. *NlSP1* transcription was analyzed by qRT-PCR with primers shown in **Supplementary Table S4**. NlSP1 protein expression was detected by immunoblotting using anti-NlSP1 antibody.

To analyzed the effect of the knockdown of NlSP1 on BPH survival rates, third-instar BPH nymphs after injection with dsRNA of *NlSP1* or *GFP*, or nuclease-free water were placed on a 1-month-old Nipponbare rice plant for every 10 nymphs. The number of surviving BPHs on each plant was recorded daily for 10 days. The experiment was repeated five times. To measure the effect of the knockdown of NlSP1 on BPH feeding, newly emerged brachypterous female adults at 3 days after the injection of *NlSP1-* or *GFP-dsRNA* or nuclease-free water into fifth-instar nymphs were using analysis of BPH growth rates. After weighing, the treated BPHs were placed into small parafilm bags (2×2.5 cm), which were then fixed on the basal stem of 1-month-old Nipponbare rice plants. After feeding 72 h, each BPH was reweighed, and the weight change of BPH served as BPH weight gain. The experiment was repeated 10 times per group, and the experiments were conducted three times.

To constitutively express *NlSP1-dsRNA* in rice, the *NlSP1*-RNAi construct described above was transformed into Nipponbare rice plants to generate the RNAi plants using an *A. tumefaciens*-mediated method. Nineteen independent transgenic T0 plants were obtained. Integration of target DNA fragments in T0 plants was determined by PCR and DNA gel-blot analysis. T0 transgenic lines SR1 and SR3 were selected for further analysis. *NlSP1-dsRNA* expression in T1 transgenic plants of SR1 and SR3 was evaluated by qRT-PCR with primers shown in **Supplementary Table S4**. The survival rates of BPHs on T1 transgenic plants of SR1 and SR3 with the highest expression level of *NlSP1-dsRNA* and wild-type plants (cv Nipponbare) were determined by releasing 10 third-instar nymphs onto each plant. The number of surviving BPHs on each plant was recorded daily for 10 days. The experiment was replicated 10 to 11 times.

### Defense Gene Expression Analyses of *N. benthamiana* Leaves and Rice Protoplasts

To detect the effect of NlSP1 on *N. benthamiana* defense-related gene expression, Constructs with NlSP1 or control GFP were transferred to *N. benthamiana* leaves mediated by *A. tumefaciens* for transient expression. Total RNA was isolated from N. benthamiana leaves 24 h and 48 h after infiltration using an EASYspin tissue/cell RNA rapid extraction kit (Yuanpinghao Biotech) according to the suppliers instructions. cDNA was synthesized using the method as described above. The expression of defense-related genes *NbPR1*, *NbPR3* and *NbPR4* was detected by qRT-PCR with primers shown in **Supplementary Table S4**. SYBR Green qRT-PCR assays were conducted as described previously. Each experiment was repeated in three independent biological replications.

To test the induction of rice protoplasts defense-related gene expression by NlSP1, Total RNA was isolated from rice protoplasts 12 h and 24 h after transformation of constructs with NlSP1 or control GFP using an EASYspin tissue/cell RNA rapid extraction kit (Yuanpinghao Biotech) according to the manufacturer’s instructions. cDNA was synthesized using the method as described above. The expression of defense-related genes *OsPR1*, *OsPR3* and *OsPR4* was detected by qRT-PCR with primers shown in **Supplementary Table S4**. SYBR Green qRT-PCR assays were conducted as described previously. Each experiment was repeated in three independent biological replications.

### Expression of NlSP1 in Yeast and Extraction and Immunoblotting of Yeast Protein

For the expression of NlSP1 in yeast, the recombinant vector NlSP1: pGBKT7-GW was transformed into yeast strain AH109 by using the Matchmaker Yeast Transformation System 2 (Clontech) according to the manufacturer’s protocol. Yeast protein was extracted by post - alkaline extraction method for western blot analysis, as detailed in the previous study (Hu et al., 2017). Yeast protein extract was separated by 10% SDS-PAGE gels. Immunoblotting was performed with anti-Myc antibody (MBL, Japan) at a dilution ratio of 1:1,000 with dilution buffer described above followed by HRP-conjugated goat anti-mouse antibodies (1:7,500 dilution). The HRP signals were detected using an Immobilon western chemiluminescent HRP substrate kit (Millpore).

### Data Analysis

Data between treatments were determined by ANOVA (Student’s t test or Tukey’s honestly significant difference test). All tests carried out with IBM SPSS Statistics version 22.

## Results

### Proteome Analysis and *In Planta* Functional Assays of Secreted Salivary Proteins Identify Candidate Effectors From *N. lugens*

**Figure 1:**
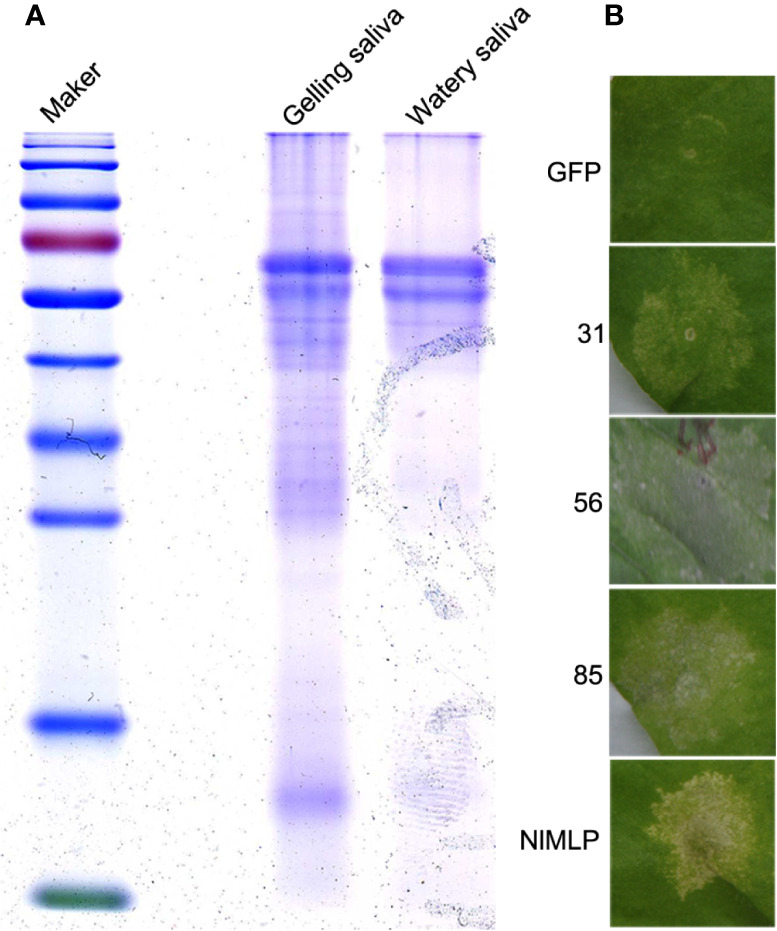
Detection of secreted saliva proteins of *N. lugens* and cell death assays in *N. benthamiana* leaves. **(A)** Coomassie Brilliant blue staining of concentrated saliva protein samples. Marker, protein marker; Gelling, gelling saliva protein; Watery, watery saliva protein. **(B)** Candidate saliva proteins of BPH induce cell death in *N. benthamiana* leaves infiltrated with *Agrobacterium tumefaciens* carrying GFP, NlMLP and three candidate proteins. The leaves were photographed 5 d after agroinfiltration. GFP, NlMLP and three candidate BPH effectors were transiently expressed with pEarleyGate100 vectors. GFP is a negative control. NlMLP is a positive control that induces cell death (Shangguan et al., 2018).

During the feeding process, BPHs repeatedly secrete both gelling and watery saliva from their salivary glands into plant cells, which plays a crucial role in plant-insect interactions. We collected BPHs’ watery and gelling saliva from a sucrose diet which 3rd instar BPH nymphs had fed upon through a membrane of stretched parafilm. SDS-PAGE analysis revealed that proteins of watery and gelling saliva exhibited similar expression patterns (**Figure 1A**). The mixed salivary proteins were subjected to shotgun LC−MS/MS analysis. Mass spectrometry data searched against the transcriptome databases of whole body and salivary glands of *N. lugens* (Liu et al., 2016; Rao et al., 2019) resulted in the identification of a salivary proteome containing 116 secreted proteins (**Supplemental Table S1**).

It has been shown pathogen effector proteins can induce cell death in non-host plants (Wroblewski et al., 2009). Previously, secretome analysis and *in planta* expression of BPH salivary proteins have identified candidate effectors that induce cell death (Rao et al., 2019). To further comprehensively explore candidate effector proteins of *N. lugens*, we combined salivary proteome identified in this study (**Supplemental Table S1**) with previously reported datum—BPH salivary proteome (Huang et al., 2016) and watery salivary proteome (Liu et al., 2016), and excluded those have been studied by secretome analysis (Rao et al., 2019). As a result, a total of 90 salivary proteins which have the most potential to be effectors involved in rice-BPH interactions were selected (**Supplemental Table S2**). We obtained full-length cDNA sequences of the candidate genes through RACE and RT-PCR, and cloned them into pEarleyGate100 vector *via* BP and LR reactions. For those candidates containing a predicted secretory signal peptide, two plasmids were constructed: one contained an open reading frame with the predicted signal peptide (ORF+SP); the other contained truncated open reading frame without the predicted signal peptide (ORF-SP). These candidates constructs were transiently expressed in *N. benthamiana* leaves *via* agroinfiltration to screen for those salivary proteins that induce cell death. The results showed three of them induced cell death (**Figure 1B**). The candidate protein numbered 85 was selected for further functional characterization and named Salivary Protein 1 (NlSP1).

### NlSP1 Induces Cell Death *In Planta*

**Figure 2:**
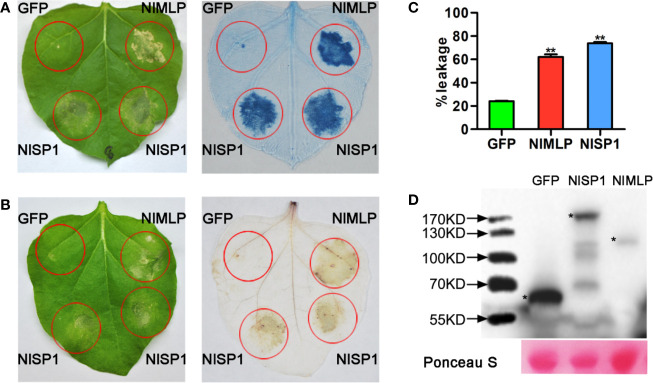
NlSP1 induces cell death in *N. benthamiana* leaves. **(A, B)** Leaves of *N. benthamiana* were infiltrated with *A. tumefaciens* carrying GFP, NlMLP and NlSP1. The leaves were photographed 3days after agroinfiltration (left) and the treated leaves were stained with Trypan blue **(A)** and DAB **(B)**. NlMLP and GFP were used as positive and negative controls, respectively. **(C)** Quantification of cell death by measuring electrolyte leakage in *N. benthamiana* leaves. Electrolyte leakage from the infiltrated leaf discs was measured as a percentage of leakage from boiled discs 4 days after agroinfiltration. Data represent means ± SE of four repeats. Asterisks above the columns indicate significant differences compared with GFP (**P < 0.01, Student’s t-test). **(D)** *N. benthamiana leaves* were harvested 2 days after agroinfiltration for immunoblot analysis with the anti-HA antibody. Asterisks indicate specific bands detected by immunoblotting analysis. Ponceau S, staining of the Rubisco large subunit was used to demonstrate loading control.

NlSP1 was recombined into the pEarleyGate101 vector (with a C-terminal YFP-HA epitope tag) for the following assays in *N. benthamiana* leaves and rice protoplasts. We verified the performance of NlSP1 to induce cell death in *N. benthamiana* leaves by Trypan blue staining. NlMLP, an effector secreted by BPH that induces HR cell death in *N. benthamiana* leaves and rice protoplasts (Shangguan et al., 2018), was used as a positive control, while GFP was used as a negative control. *N. benthamiana* leaves expressed in NlSP1 can be dyed dark blue (**Figure 2A**), suggesting that NlSP1 triggered a strong cell death in *N. benthamiana* leaves. Moreover, ion leakage of leaves expressing NlSP1 was significantly higher than that of leaves expressing GFP (**Figure 2C**). Immunoblot analysis confirmed these fusion proteins were properly expressed in *N. benthamiana* leaves (**Figure 2D**).

To test whether NlSP1 induces cell death in the host plant, we transiently expressed NlSP1 in rice protoplasts with positive control NlMLP and negative control GFP. Fluorescein diacetate (FDA) staining of the protoplasts showed that the cell viability of protoplasts expressing NlSP1 or NlMLP was significantly lower than that of control protoplasts expressing GFP (**Supplemental Figures S1A, B**). We also coexpressed NlSP1 together with the luciferase (LUC) gene in rice protoplasts. LUC activity was significantly lower in protoplasts coexpressing NlSP1 compared with the negative control coexpressing GFP (**Supplemental Figure S1C**). Taken together, the results indicated that NlSP1 could induce cell death *in planta*.

### NlSP1 Activates Plant Defense Responses

**Figure 3:**
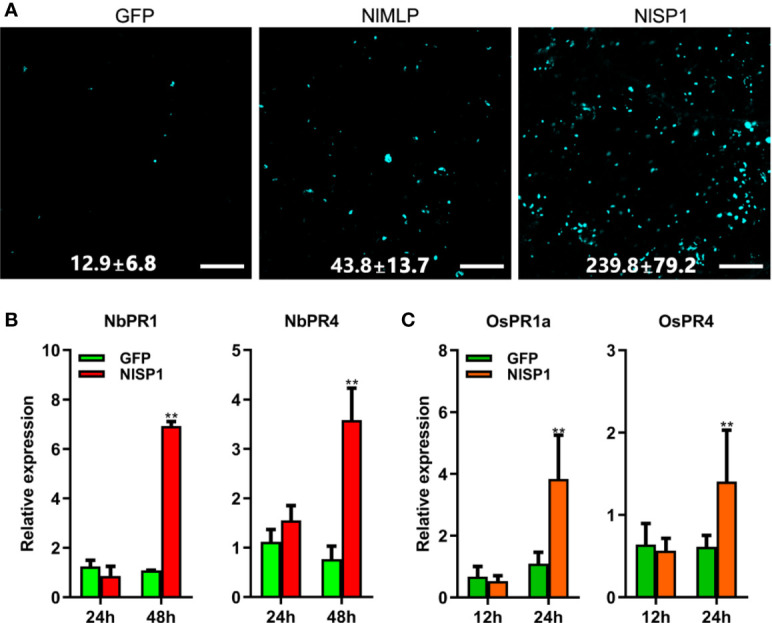
NlSP1 activates defense responses in *N. benthamiana* and rice protoplasts. **(A)** *N. benthamiana* leaves infected with agrobacterium containing GFP, NlMLP, and NlSP1 were sampled 48 h after inoculation, stained with aniline blue and photographed using a fluorescence microscope. Numbers indicate means ± SD of callose spots obtained from 3 individual leaf discs. Scale bar = 100 um. **(B)** Expression analysis of pathogenesis-related genes *NbPR1* and *NbPR4* in *N. benthamiana* leaves after instantaneous transformation of GFP or NlSP1 at 24 h and 48h. Data represent means ± SD of three repeats. Asterisks above the columns indicate significant differences compared with GFP (**P < 0.01; Student’s t-test). **(C)** Expression analysis of defense-related genes *OsPR1a* and *OsPR4* in rice protoplast after transformation of GFP or NlSP1 at 12 h and 24 h. Data represent means ± SD of three repeats. Asterisks above the columns indicate significant differences compared with GFP (**P < 0.01; Student’s t-test).

ROS burst, callose deposition, and activation of JA and SA signaling pathways are hallmarks of plant defense responses against injury by insect pests or pathogens (Walling, 2000; Hao et al., 2008; Ge et al., 2015; Zhao et al., 2016). We transiently expressed NlSP1 in *N. benthamiana* leaves or rice protoplasts to investigate reactive oxygen species (ROS) generation, callose deposition, and defense gene expression. The transformed *N. benthamiana* leaves were stained with 3,3′-diaminobenzidine (DAB) to detect the content of hydrogen peroxide (**Figure 2B**). Regions expressing NlSP1 in *N. benthamiana* leaves were stained dark yellow by DAB, but regions expressing GFP did not, indicating that NlSP1 caused the accumulation of hydrogen peroxide in *N. benthamiana* leaves. *N. benthamiana* leaves expressing NlSP1 also showed stronger callose deposition than leaves expressing GFP as revealed by Aniline Blue staining (**Figure 3A**). According to callose spots count statistics, callose deposition induced by NlSP1 was 20 times higher than that of GFP, which showed a very significant difference.

We also analyzed defense-related gene expression. The relative expression of the SA-related marker genes *Pathogenesis Related 1* (*PR1*), and the JA-related marker genes *Pathogenesis Related 4* (*PR4*) in *N. benthamiana* leaves were determined by quantitative reverse transcription (qRT)-PCR at 1 and 2 days post infiltration. NlSP1 induced transcriptional activation of *NbPR1* and *NbPR4* (**Figure 3B**). The similar results were found in rice protoplasts that transiently expressed NlSP1 (**Figure 3C**). Immunoblot analysis confirmed the expression of NlSP1 in rice protoplasts (**Supplemental Figure S2**). Taken together, NlSP1 activated plant defense responses by inducing ROS generation, callose deposition, and PR genes expression *in planta*.

### The Characterization of NlSP1

**Figure 4:**
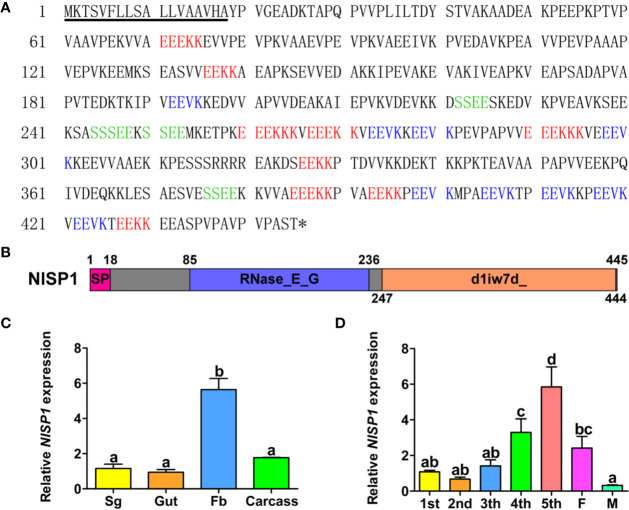
The Characterization of NlSP1. **(A)** Amino acid sequence of the NlSP1. The solid underline indicates the predicted signal peptide as predicted by SignalP-5.0. The asterisk (*) indicates the stop codon. The different short repeat regions are indicated by different colors. **(B)** Protein domain analysis of NlSP1. The pink box served as the predicted signal peptide, the blue served as the predicted domain RNase_E_G, and the orange served as the predicted domain d1iw7d_. **(C)** Expression patterns of NlMLP gene in different tissues of BPH. (Sg, salivary gland; Fb, fat body). Normalized against *β-actin* gene expression, determined by qRT-PCR. Data represent means ± SD of three repeats, N = 30. Different letters above the bars indicate significant differences, as determined by a Tukey honest significant difference test (P < 0.05). **(D)** Expression patterns of NlMLP gene in developmental stages of BPH. (1st to 5th, 1st to 5th instar; F, female adult; M, male adult), normalized against β-actin gene expression, determined by qRT-PCR. Data represent means ± SE of three repeats. N = 30. Different letters above the bars indicate significant differences, as determined by a Tukey honest significant difference test (P < 0.05).

*NlSP1* contains a 1,338-bp open reading frame and encodes a peptide containing 445 amino acid residues with a predicted molecular weight (MW) of 48.6 kDa and a pI of 4.79 (accession no. MT459811; **Figure 4A**). The first 18 amino acids at the N-terminal of NlSP1 make up the predicted signal peptide, with cleavage predicted between residues 18 and 19. Moreover, NlSP1 protein contains several short repeats (EEKK, EEVK, SSEE; **Figure 4A**). NlSP1 does not contain cysteine, the basic amino acid that forms disulfide bonds (**Figure 4A**).We found no NlSP1 homologous protein by NCBI BLAST. Combined with the predicted results of NCBI and SMART, NlSP1 protein contains two domains: one RNase_E_G superfamily domain located in amino acids 85–236 (E-value = 2.56e-08), and the other SCOP d1iw7d_ domain located in amino acids 247-444 (E-value = 1.30e-02; **Figure 4B**).

To investigate the functions of NlSP1, we analyzed NlSP1 mRNA levels in different tissues, including salivary glands, midguts, fat bodies, and the remaining parts, *via* qRT-PCR. The results showed NlSP1 was highly expressed in the fat bodies, which was significantly different from the other three groups (**Figure 4C**). We also analyzed the expression of NlSP1 in BPHs at various developmental stages, including the nymphs of first to fifth instar, female and male adults. The expression of NlSP1 was significantly increased during the growth stage of BPH nymphs, and reached the highest expression level at the fifth instar nymph stage (**Figure 4D**). These results suggested NlSP1 might play an essential role in the growth and development of BPH.

**Figure 5:**
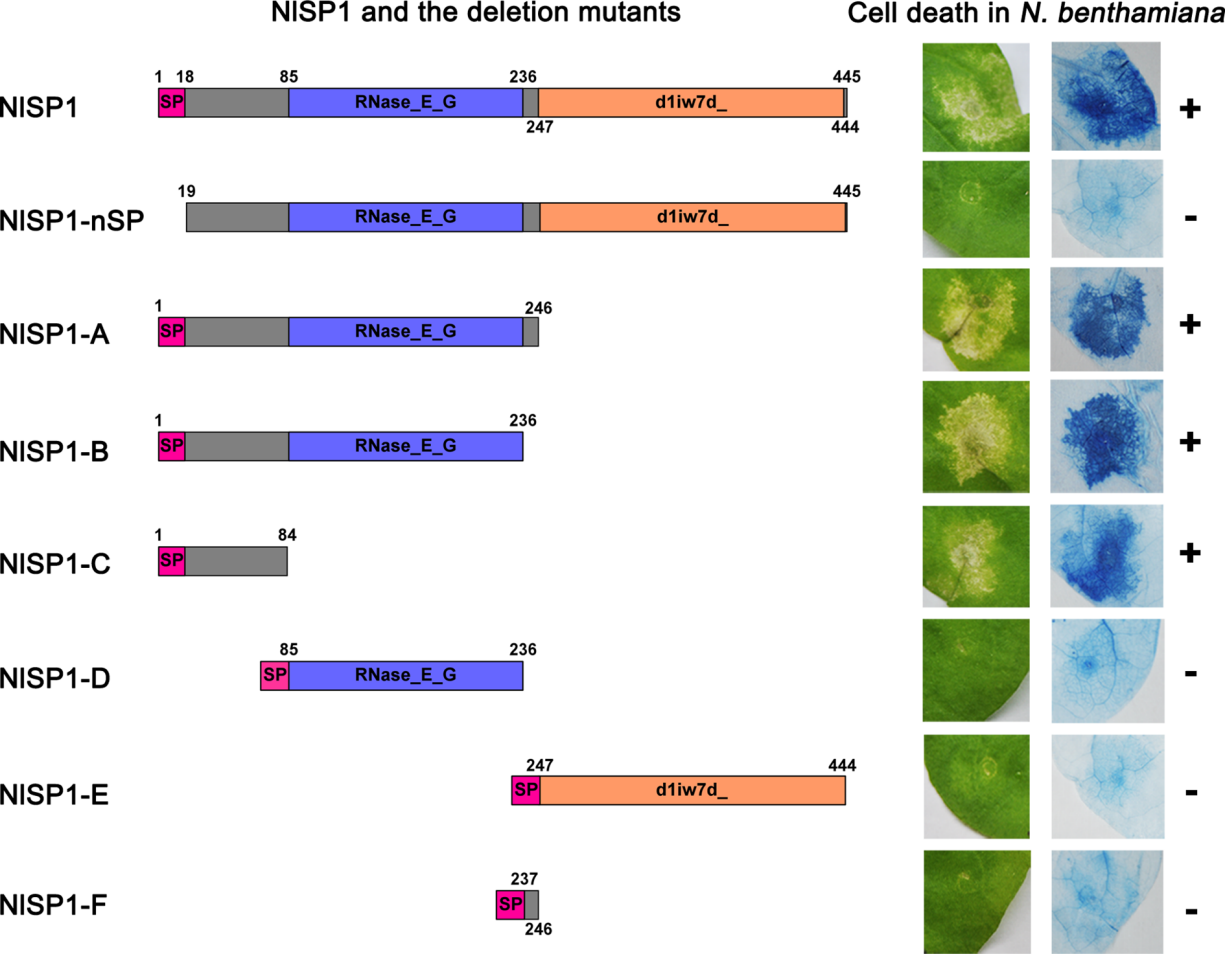
Deletion analysis of NlSP1. Left column, schematic views of NlSP1 and tested deletion mutants. SP, the predicted signal peptide. The number around the box indicates the amino acid position of NlSP1. Middle column: Cell-death lesions on *N. benthamiana* leaves expressing NlSP1 deletion mutants. Right column: corresponding Trypan blue staining results of *N. benthamiana* leaves. The + and – signs indicate obvious cell death and no cell death, respectively. Each experiment was repeated at least three times with same results.

To determine the functional domains of NlSP1 required for its cell death induction activity, we generated a series of NlSP1 deletion mutants and analyzed them by the way of cell death assays (**Figure 5**). The predicted signal peptide deletion mutant NlSP1-nSP did not trigger cell death in *N. benthamiana* leaves, indicating that the predicted signal peptide is necessary for NlSP1 to induce cell death. The C-terminal deletion mutants NlSP1-A, B, and C triggered cell death in *N. benthamiana* leaves, while the other deletion mutants NlSP1-D, E, and F did not (**Figure 5**). Further Trypan blue staining was performed as a revalidation of the cell death phenotype (**Figure 5**). Protein immunoblotting revealed these mutant proteins were properly expressed (**Supplemental Figure S3**). These results demonstrated that N-terminal 1–84 amino acid region of NlSP1 is required for its cell death induction activity.

### NlSP1 Protein Can Be Secreted Into Rice and Form Complex With Certain Interacting Partner of Rice

**Figure 6:**
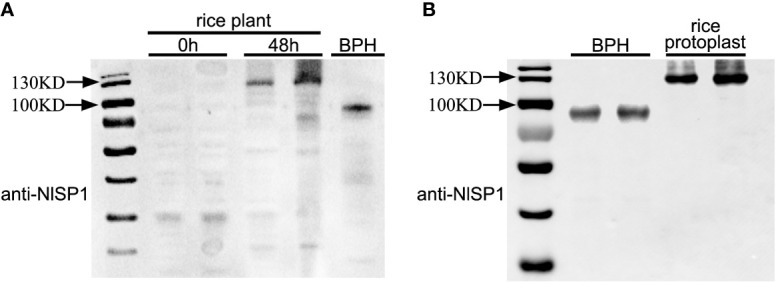
NlSP1 protein can be secreted into rice and form complex with certain protein of rice interacting partner of rice. **(A)** NlSP1 can be secreted into rice. 0h and 48h, the protein samples from leaf sheath of rice plants after BPH feeding at 0h and 48h; BPH, the protein sample from the whole body of BPHs. All protein samples were detected by Western blot with the anti-NlSP1 antibody. Asterisks indicate specific bands detected by immunoblotting analysis. **(B)** Expression of NlSP1 in different species for immunoblotting analysis with the anti-NlSP1 antibody. Rice, protein samples of NlSP1 expressed in rice protoplasts; BPH, protein samples of NlSP1 expressed in *Nilaparvata lugens*.

NlSP1 was identified from BPH salivary proteome, suggesting that it could be secreted into rice tissues during BPH feeding. To test this possibility, we extracted proteins from the leaf sheaths of plants following BPH feeding and performed immunoblot analysis using anti-NlSP1 antibodies. NlSP1 was detected in protein extracts from BPH-infested rice plants, but not in protein extracts from noninfested control plants (**Figure 6A**), demonstrating that NlSP1 is secreted into rice tissues during BPH feeding.

*NlSP1* encodes a peptide containing 445 amino acid residues with a predicted MW of 48.6 kDa. Interestingly, we noticed that, in western blot, NlSP1 protein in BPHs migrated as a protein of approximately 100 kDa in SDS-PAGE gel, approximately twice larger than its predicted MW (**Figure 6A**). More importantly, when NlSP1 was secreted into rice tissues, it migrated at ~130 kDa, even larger than that in BPHs (**Figure 6A**). To validate this result, NlSP1 construct without any epitope tag was transiently expressed in rice protoplasts. Protein extracts were separated by SDS-PAGE and subjected to immunoblotting using anti-NlSP1 antibodies. Immunoblotting showed the apparent molecular mass of NlSP1 protein expressed in rice protoplasts was about 130 kDa (**Figure 6B**), identical to that of NlSP1 protein secreted into rice plants (**Figure 6A**), indicating NlSP1 protein might have undergone unknown post-translational modifications (PTMs) or formed a complex with certain interacting partner of rice.

Actually, NlSP1 protein also exhibited drastic variations from its predicted MW when expressed in *N. benthamiana* (**Figure 2D** and **Supplemental Figure S3**), yeast (**Supplemental Figure S4A**) and *Escherichia coli* (**Supplemental Figures S4B, C**), as revealed by Immunoblotting. Recombinant NlSP1 protein expressed in *Escherichia coli* detected with anti-HIS and anti-NlSP1 antibodies showed the identical results (**Supplemental Figures S4B, C**), confirming the specificity of the prepared anti-NlSP1 antibody. These results also suggested that the MW difference observed is attributed to the activity/functionality conferred by NlSP1 protein.

### NlSP1 Localizes to the Cytoplasm of Rice Cells

**Figure 7:**
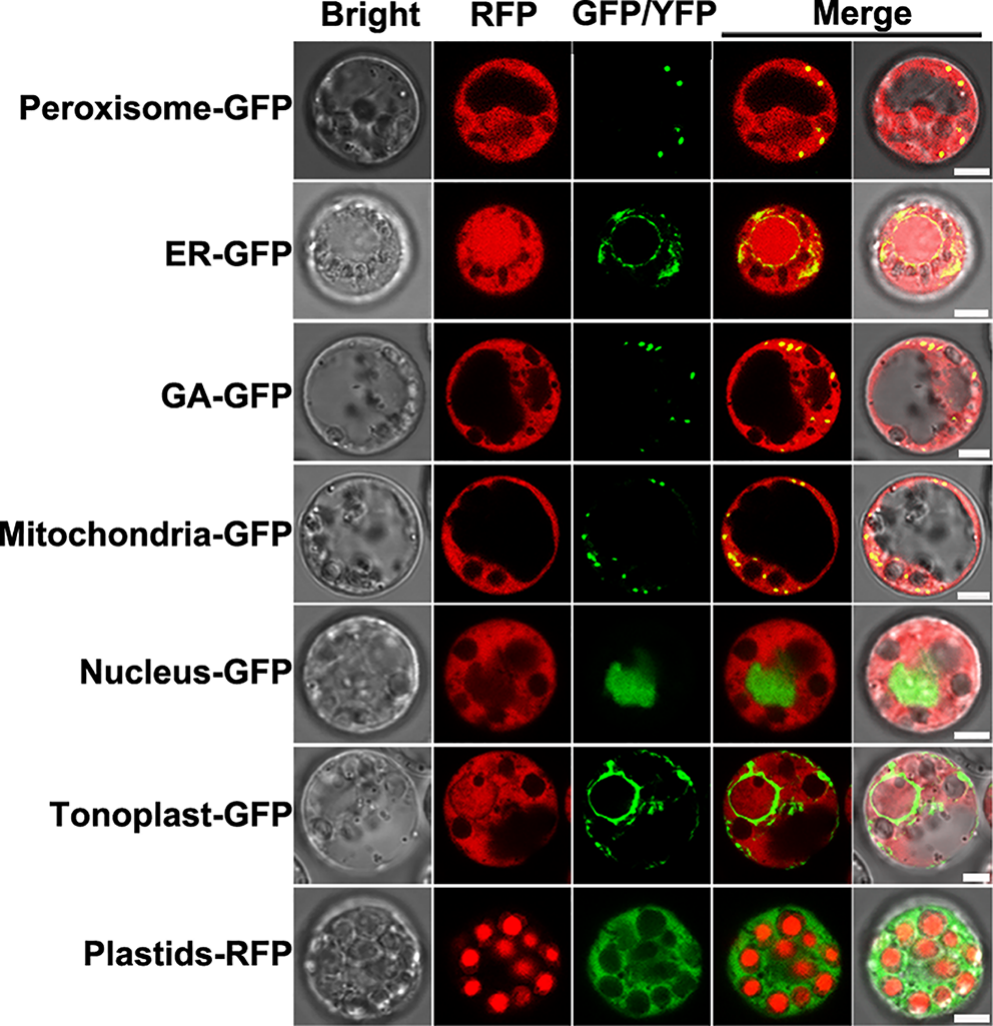
Subcellular localization of NlSP1 in rice cells. NlSP1-RFP was co-transformed with peroxisome marker (CD3-979, FP-PTS1), ER marker (CD3-955, AtWAK2-HDEL), GA marker (CD3-963, Man49), mitochondrial marker (CD3-987, ScCOX4), nuclear marker (bZIP63), and tonoplast marker (CD3-971, γ-TIP). NlSP1-YFP was transformed in protoplast of rice, and Plastids-RFP served as spontaneous fluorescence of plastids. Scale bar = 5 μm.

As described above, NlSP1 protein can be secreted into rice. In order to determine where the NlSP1 functions in rice cells, we conducted localization experiments using rice protoplasts. NlSP1-RFP fusion gene and organelle markers were transiently co-expressed in rice protoplasts and their co-localization was observed under a confocal laser scanning microscopy. NlSP1 can co-locate with a range of organelles, including peroxisome, endoplasmic reticulum (ER), Golgi apparatus (GA), and mitochondrial, but it cannot co-locate with nuclear and tonoplast (**Figure 7**). In addition, by comparing the fluorescence of NlSP1-YFP fusion protein expressed in rice protoplasts with the autofluorescence of the plastids, we found NlSP1 was not co-located with the plastids. These results suggested NlSP1 localizes to the cytoplasm of rice cells.

### NlSP1 Is Required for BPH Feeding and Survival

**Figure 8:**
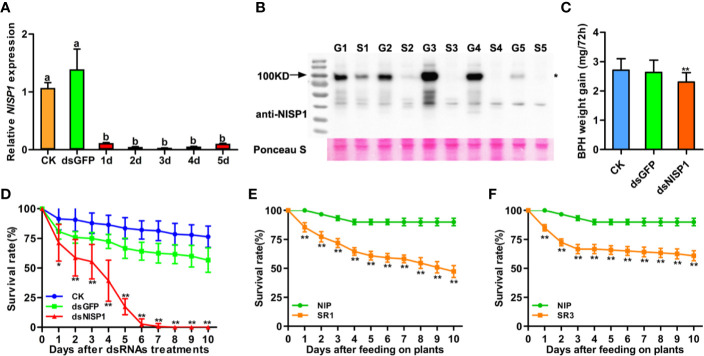
Effects of *NlSP1* silencing on BPH feeding and performance. **(A)** Relative levels of *NlSP1* expression after microinjection of dsRNA were normalized against *β-actin* gene expression, determined by qRT-PCR. CK, BPHs injected with DEPC H_2_O; dsGFP, BPHs injected with GFP-dsRNA; d1-d5, 1 to 5 days of BPHs injected with NlSP1-dsRNA. Data represent means ± SE of 4 repeats. Different letters above the bars indicate significant differences, as determined by a Tukey honest significant difference test (P < 0.05). **(B)** Western blot detection of NlSP1 protein after microinjection of dsRNA. G1-G5, BPHs injected with GFP-dsRNA in 1 to 5 days; S1-S5, BPHs injected with NlSP1-dsRNA in 1 to 5 days. Western blot detection using anti-NlSP1 antibody. Asterisks indicate specific bands detected by immunoblotting analysis. Ponceau S served as loading control. **(C)** BPH gain weight of BPHs feeding on Nipponbare rice after injection of CK, dsGFP or dsNlSP1. Data represent means ± SD of 15 independent experiments. Asterisks above the bars indicate significant differences compared with CK (*P < 0.05; **P < 0.01; Student’s t-test). **(D)** The survival rate of BPH feeding on Nipponbare rice after injection monitored daily. CK, BPHs injected with DEPC H_2_O; dsGFP, BPHs injected with GFP-dsRNA; dsNlSP1, BPHs injected with NlSP1-dsRNA. The experiment was repeated 10 times, with 10 BPHs. Data represent means ± SE of 10 repeats. Asterisks indicate significant differences compared with CK (*P < 0.05; **P < 0.01; Student’s t-test). **(E)** The survival rate of BPH feeding on NlSP1-RNAi T1 transgenic plants SR1 and WT plants. NIP, wild-type plant Nipponbare; SR1, an independent transgenic line expressing *NlSP1-dsRNA*. T1 transgenic plants of SR1 with the highest expression level of *NlSP1-dsRNA* were used for BPHs survival rate analysis. Data represent means ± SE of 11 independent experiments. Asterisks indicate significant differences compared with NIP (**P < 0.01; Student’s t-test). **(F)** The survival rate of BPH feeding on NlSP1-RNAi T1 transgenic plants SR3 and WT plants. NIP, wild-type plant Nipponbare; SR3, an independent transgenic line expressing *NlSP1-dsRNA*. T1 transgenic plants of SR3 with the highest expression level of *NlSP1-dsRNA* were used for BPHs survival rate analysis. Data represent means ± SE of 10 independent experiments. Asterisks indicate significant differences compared with NIP (**P < 0.01; Student’s t-test).

To explore the function of NlSP1 in BPH, we injected double-stranded RNA (dsRNA) of NlSP1 into third instar BPH nymphs to mediate RNA interference (RNAi; Liu et al., 2010). Compared with the two control groups receiving either no injection or injection with dsGFP, the mRNA levels of *NlSP1* in the whole body of BPH injected with dsNlSP1 were significantly reduced to less than 10% from the first day after microinjection, and the silencing effect lasted for more than 5 days (**Figure 8A**). Injection of dsNlSP1 also reduced the abundance of NlSP1 protein in BPH to undetectable level as revealed by immunoblotting (**Figure 8B**). The treated BPH insects were allowed to feed on Nipponbare rice plants. Compared to the two control groups, BPH insects injected with dsNlSP1 had a significantly lower survival rate from 2 to 10 d after microinjection, which were almost completely dead at 6th days after microinjection (**Figure 8D**). The similar results were obtained when BPH injected with dsNlSP1 were fed on artificial diets (**Supplemental Figure S5**), indicating silencing of NlSP1 has a lethal effect on BPH. BPHs subjected to dsNlSP1 treatment also had significantly smaller weight gain values, an indicator of food intake dose, than the two control groups (**Figure 8C**), indicating silencing of NlSP1 reduces feeding ability of BPH.

Double-Stranded RNA technology to control insect pests is a promising new control strategy in the past decade, which can silence the necessary genes of pests and lead to toxic effects (Zha et al., 2011; Christiaens et al., 2020). We transformed BPH-susceptible rice plants with *NlSP1-dsRNA* to further verify the role of NlSP1 in BPH. Nineteen independent transgenic T0 plants were obtained and two representative lines SR1 and SR3 were selected for further analysis. The expression levels of *NlSP1-dsRNA* were detected in T1 transgenic positive plants of SR1 and SR3, and the plants with the highest expression level of *NlSP1-dsRNA* were used for BPHs survival rate analysis (**Supplemental Figures S6A, B**). BPHs fed on SR1 and SR3 T1 transgenic positive plants had a significantly lower survival rates than that of BPHs fed on wild-type Nipponbare plants (**Figures 8E, F**). Taken together, these results demonstrate that NlSP1 is essential for BPH feeding and survival.

## Discussion

Saliva is a complex mixture of biomolecules and plays a crucial role in the feeding process of plants sap-sucking insects (Miles, 1999; Will et al., 2013). Not only does it contain a suite of bioactive compounds that regulate the inhibition or bypassing of plant defenses, enabling insects to successfully detect plants and ingest their juices, but it also contains PAMPs and effectors that induce plant defenses (De Vos and Jander, 2009; Sharma et al., 2014; Stahl et al., 2018). Rao et al. (2019) sequenced the salivary gland transcriptomes of BPH and established a secretome composed of 1,140 conserved or rapidly evolving salivary proteins. Six were identified as candidate effector proteins that elicit defense responses through transient expression analysis in *N. benthamiana* leaves. In this study, we collected BPHs’ watery and gelling saliva and identified the salivary proteome (**Supplemental Table S1**). Together with previously reported BPH salivary proteome (Huang et al., 2016) and watery salivary proteome (Liu et al., 2016), these salivary proteins identified represent a large effector repertoire involved in the interaction between BPH and rice. *In planta* functional assays of these secreted salivary proteins have identified three candidate effectors that induce cell death (**Figure 1B**). Salivary Protein 1 (NlSP1) was further characterized in this study.

NlSP1 is unique to BPH and has typical amino acid tandem duplication, which is consistent with previous analyses (Rao et al., 2019). NlSP1 protein contains a RNase_E_G superfamily domain and a SCOP d1iw7d_ domain. It has been shown that RNase E or RNase G can form dimers or tetramers by the interface between the large and small domains and the zinc bond between the structural zinc ions (Aït-Bara and Carpousis, 2015). As revealed by immunoblotting, NlSP1 protein in BPHs migrated as approximately twice larger than its predicted MW (**Figure 6A**). We demonstrated that the salivary protein NlSP1 can be secreted into rice tissues. More importantly, when NlSP1 was secreted into rice tissues or transiently expressed in rice protoplasts, it migrated at ~130 kDa, approximately 30 kDa larger that of in BPHs (**Figures 6A, B**). Similar phenomena were also observed when NlSP1 protein was expressed in *N. benthamiana* (**Figure 2D** and **Supplemental Figure S3**), yeast (**Supplemental Figure S4A**) and *Escherichia coli* (**Supplemental Figures S4B and C**). These results indicated that NlSP1 protein might have undergone unknown PTMs or formed a complex with certain interacting partner. As a kind of HAMPs, a fatty acid–amino acid conjugate (FAC) volicitin and other FACs in herbivore oral secretion are formed by an insect-derived amino acid and a plant-derived fatty acid (Stahl et al., 2018). When implemented to cowpea or maize, the plant-derived protein fragment inceptin induces various defense responses, including the production of volatiles, defense-related hormones, and defense compounds (Schmelz et al., 2006). Thus, we can reasonably speculate that PTMs of NlSP1 or the complex formed by BPH-derived NlSP1 protein with certain interacting partner of rice plant might play a critical role in rice-BPH interaction, which needs a further investigation.

The defense reaction elicited by NlSP1 shares common features with immune responses shown by well-known effectors and pathogen-associated molecular patterns (Shangguan et al., 2018). When transiently expressed in *N. benthamiana* leaves and rice protoplasts, NlSP1 triggers cell death, which is a common phenomenon in effector-triggered immune responses (Rao et al., 2019). By DAB staining, H_2_O_2_ accumulation was found in tobacco leaves expressing NlSP1 (**Figure 2B**), indicating that NlSP1 can induce ROS. Excessive accumulation of ROS was known to cause cell death in transfected areas (Mur et al., 2008). We conjectured that the cell death induced by NlSP1 is caused by the accumulation of ROS. *NbPR4* encodes a hevein-like chitinase and is a JA pathway marker gene (Kiba et al., 2014). *NbPR1* be induced by SA and the up-regulation of is a characteristic feature of the activated SA-signaling pathway (Zhu et al., 2012; Shangguan et al., 2018). We found the expression of NlSP1 induced the *NbPR4* and *NbPR1* (**Figure 3B**). Callose deposition is an effective plant response to pests and pathogens (Hao et al., 2008; Kuśnierczyk et al., 2008; Luna et al., 2011). *N. benthamiana* leaves expressing NlSP1 also showed strong callose deposition (**Figure 3A**). All of these demonstrated NlSP1 activates a variety of defense responses *in planta*.

The expression pattern analysis revealed NlSP1 showed the highest expression level in fat body, an organ of great biosynthetic and metabolic activity (Keeley, 1985; Arrese and Soulages, 2010), suggesting NlSP1 may play an essential role in the growth and development of BPH. When NlSP1 expression was knocked down using a dsRNA microinjection method, BPH feeding was inhibited and insect performance was reduced significantly (**Figures 8C, D**). These results demonstrated that NlSP1 is required for BPH feeding and survival, similar to those BPH secreted salivary proteins (Ji et al., 2017; Ye et al., 2017; Shangguan et al., 2018). Double-Stranded RNA technology is a promising strategy for insect control, as the transgenic plants expressing dsRNA can effectively kill the pest population and reduce the damage to crops (Joga et al., 2016). Rice transgenic plants expressing *NlMLP-dsRNA* can impair salivary sheath formation and significantly reduce feeding ability and survival rate of BPH (Shangguan et al., 2018). We found *NlSP1-dsRNA* transgenic rice plants also significantly reduced the survival rate of BPHs (**Figure 8E, F**), making *NlSP1* an ideal target for control of this devastating insect.

In summary, our results indicate that NlSP1 may be an effector involved in rice and BPH interactions. NlSP1 is necessary for the survival of BPH and plays an important role in BPH feeding. NlSP1 protein can form a complex with certain interacting partner of rice when secreted into rice plant. NlSP1 can activate defense responses by inducing ROS generation, callose deposition, and PR genes expression *in planta*. Further studies are needed to identify rice interacting partner of NlSP1 and figure out the role of the complex. The novel molecular interacting way we found would provide new insight and direction for studying the interaction between BPH and rice.

## Data Availability Statement

All datasets presented in this study are included in the article/**Supplementary Material**.

## Author Contributions

RC, LY, and GH conceived and supervised the project. RC and JH designed the experiments. JH performed most of the experiments. NZ, JS, YP, JG, CZ, SS, XZ, DW, WG, KY, BD, LZ, and GH performed some of the experiments. JH and RC analyzed data and wrote the manuscript.

## Funding

This work was supported by grants from the National Natural Science Foundation of China (31630063) and the National Key Research and Development Program (2016YFD0100600 and 2016YFD0100900, both to GH).

## Conflict of Interest

The authors declare that the research was conducted in the absence of any commercial or financial relationships that could be construed as a potential conflict of interest.

## Supplementary Material

Figure S1 NlSP1 induces cell death in rice protoplasts. (A) Images of FDA-stained viable rice protoplasts transformed with GFP, NlMLP or NlSP1. Living cells were visualized using confocal laser-scanning microscopy and images were taken 40 h after transformation. GFP, a negative control that cannot induce cell death. NlMLP, a positive control that can induce cell death. Scale bar = 25 μm. **(B)** Numbers of FDA-stained viable rice protoplasts transformed with GFP, NlMLP or NlSP1. Means and SEs were calculated from three independent experiments, and 10 randomly selected microscopy fields were counted per experiment. Asterisks above the columns indicate significant differences compared with GFP (**, P < 0.01, Student’s t-test). **(C)** RLUC activity in rice protoplasts co-expressing LUC and NlSP1, GFP or NlMLP. Means and SEs were calculated from three independent experiments. Asterisks above the columns indicate significant differences compared with GFP (**, P < 0.01, Student’s t-test).

Figure S2 Western blot analysis of proteins from rice protoplasts transformed with GFP and NlSP1. Rice protoplasts were harvested at 16 h for immunoblotting analysis with the anti-HA antibody. Ponceau S, staining of the Rubisco large subunit served as loading control.

Figure S3 Immunoblotting of proteins from *N. benthamiana* leaves transiently expressing NlSP1 deletion mutants. *N. benthamiana* leaves were harvested 2 days after agroinfiltration for immunoblot analysis with the anti-HA antibody. Asterisks indicate specific bands detected by immunoblotting analysis.

Figure S4 Immunoblotting analysis of NlSP1 protein expressed in yeast and *Escherichia coli*. **(A)** Expression of fusion protein in yeast for immunoblotting analysis with the anti-Myc antibody. The fusion protein was GAL4-BD+Myc epitope tag+NlSP1 without the predicted signal peptide. **(B, C)** Expression of NlSP1 in *Escherichia coli* for immunoblotting analysis with the anti-His antibody (B) and with the anti-NlSP1 antibody (C). NlSP1-His, the concentrated NlSP1-His protein samples eluted with 30mM imidazole eluent.

Figure S5 The survival rate of BPH feeding on artificial diet of BPH after injection monitored daily. CK, BPHs injected with DEPC H_2_O; dsGFP, BPHs injected with GFP-dsRNA; dsNlSP1, BPHs injected with NlSP1-dsRNA. The experiment was repeated 5 times, with 10 BPHs. Data represent means ± SE of five repeats. Asterisks indicate significant differences compared with CK (**, P < 0.01; Student’s t-test).

Figure S6 The relative expression of *NlSP1-dsRNA* in two independent NlSP1-RNAi T1 transgenic plants. **(A)** The relative expression of *NlSP1-dsRNA* in T1 transgenic plants of SR1. NIP, wild-type plant Nipponbare; SR1, an independent transgenic line expressing *NlSP1-dsRNA*. SR1-1–11, 11 T1 transgenic plants of SR1. **(B)** The relative expression of *NlSP1-dsRNA* in T1 transgenic plants of SR3. SR3, an independent transgenic line expressing *NlSP1-dsRNA*. SR3-1–10, 10 T1 transgenic plants of SR3.

Table S1 Summarizes the proteins of saliva of N. lugens identified by Shotgun LC–MS/MS.

Table S2 Candidate proteins from the salivary proteome of N. lugens.

Table S3 List of PCR primers used in this study.

Table S4 List of qRT-PCR primers used in this study.
