# Supplementary figures and images for: Salivary Protein 1 of Brown Planthopper Is Required for Survival and Induces Immunity Response in Plants
Source: Front Plant Sci. 2020 Aug 27;11:571280. doi: 10.3389/fpls.2020.571280 (PMC7481525; doi:10.3389/fpls.2020.571280)

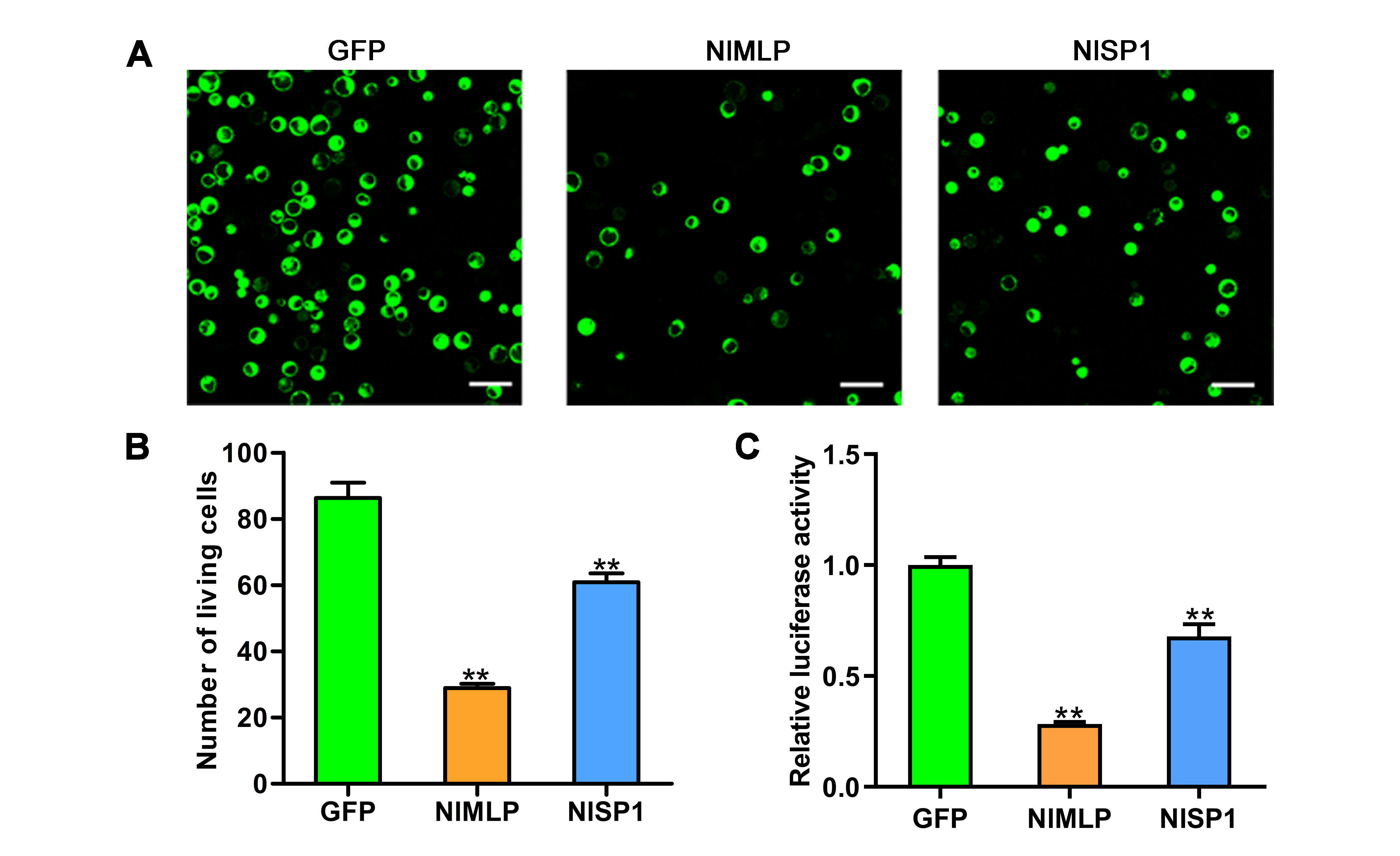

Supplement: Figure S1 — NlSP1 induces cell death in rice protoplasts. (A) Images of FDA-stained viable rice protoplasts transformed with GFP, NlMLP or NlSP1. Living cells were visualized using confocal laser-scanning microscopy and images were taken 40 h after transformation. GFP, a negative control that cannot induce cell death. NlMLP, a positive control that can induce cell death. Scale bar = 25 μm. (B) Numbers of FDA-stained viable rice protoplasts transformed with GFP, NlMLP or NlSP1. Means and SEs were calculated from three independent experiments, and 10 randomly selected microscopy fields were counted per experiment. Asterisks above the columns indicate significant differences compared with GFP (**, P < 0.01, Student’s t-test). (C) RLUC activity in rice protoplasts co-expressing LUC and NlSP1, GFP or NlMLP. Means and SEs were calculated from three independent experiments. Asterisks above the columns indicate significant differences compared with GFP (**, P < 0.01, Student’s t-test). [file Image_1.tif]

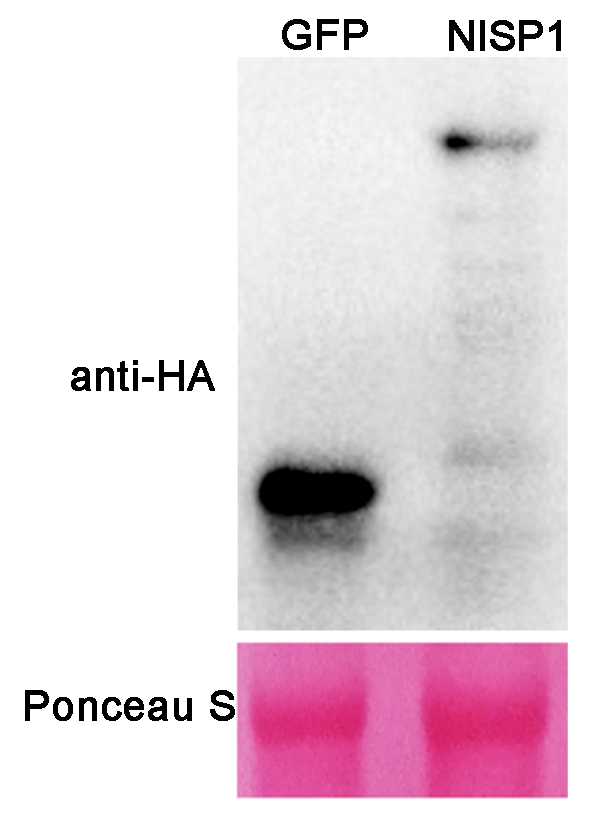

Supplement: Figure S2 — Western blot analysis of proteins from rice protoplasts transformed with GFP and NlSP1. Rice protoplasts were harvested at 16 h for immunoblotting analysis with the anti-HA antibody. Ponceau S, staining of the Rubisco large subunit served as loading control. [file Image_2.tif]

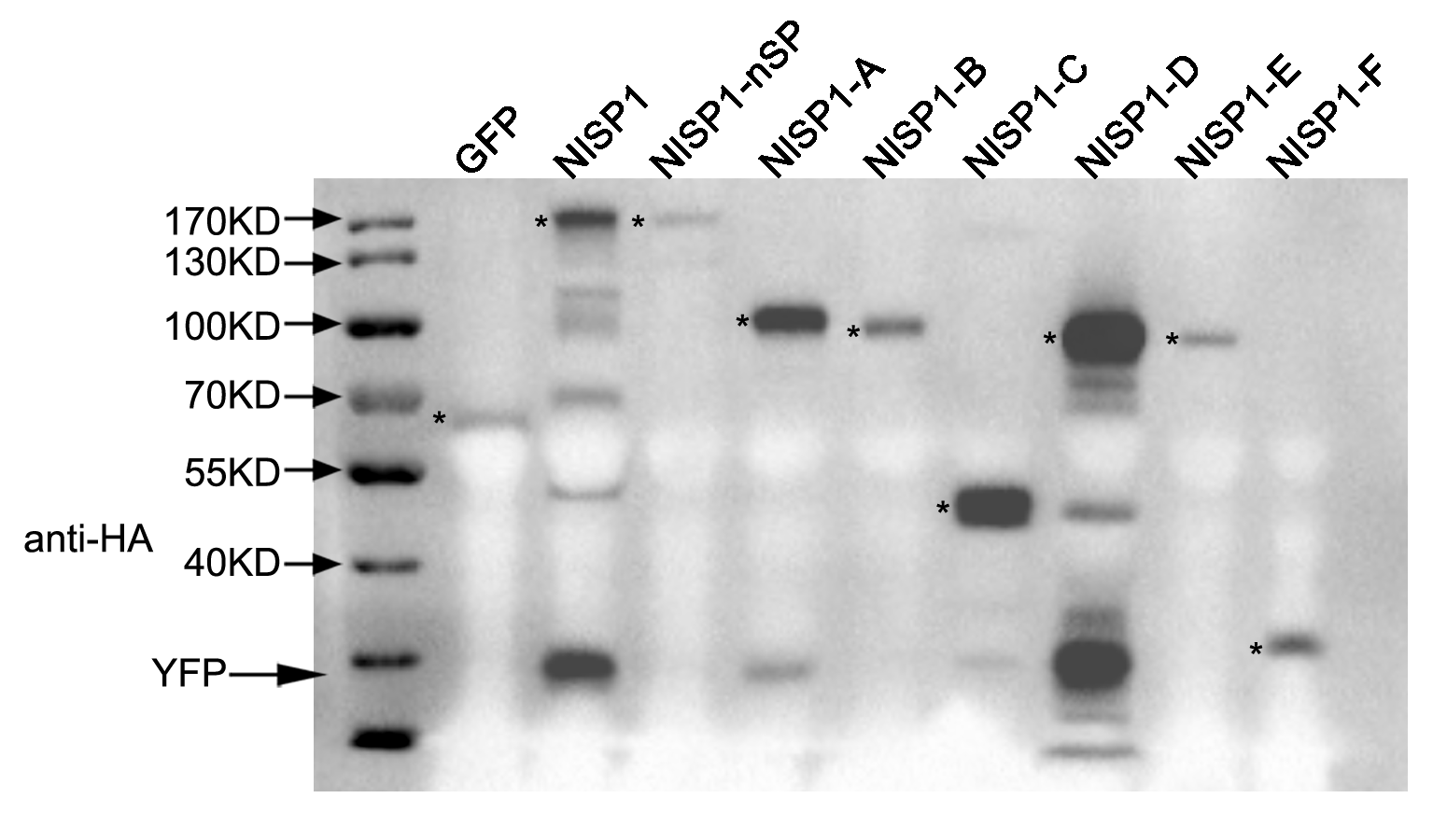

Supplement: Figure S3 — Immunoblotting of proteins from N. benthamiana leaves transiently expressing NlSP1 deletion mutants. N. benthamiana leaves were harvested 2 days after agroinfiltration for immunoblot analysis with the anti-HA antibody. Asterisks indicate specific bands detected by immunoblotting analysis. [file Image_3.tif]

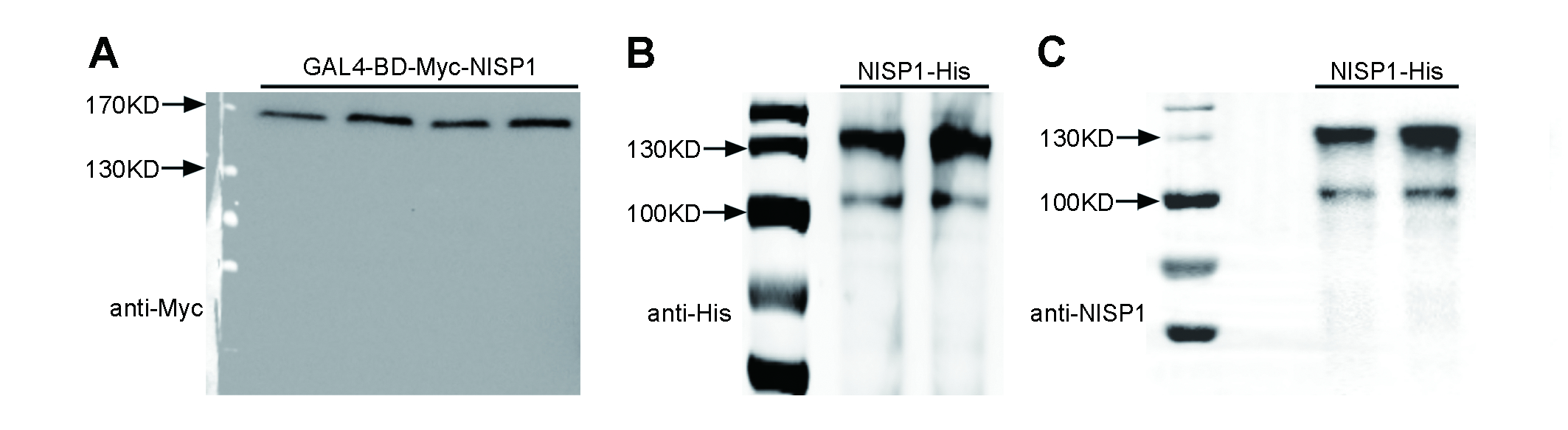

Supplement: Figure S4 — Immunoblotting analysis of NlSP1 protein expressed in yeast and Escherichia coli. (A) Expression of fusion protein in yeast for immunoblotting analysis with the anti-Myc antibody. The fusion protein was GAL4-BD+Myc epitope tag+NlSP1 without the predicted signal peptide. (B, C) Expression of NlSP1 in Escherichia coli for immunoblotting analysis with the anti-His antibody (B) and with the anti-NlSP1 antibody (C). NlSP1-His, the concentrated NlSP1-His protein samples eluted with 30mM imidazole eluent. [file Image_4.tif]

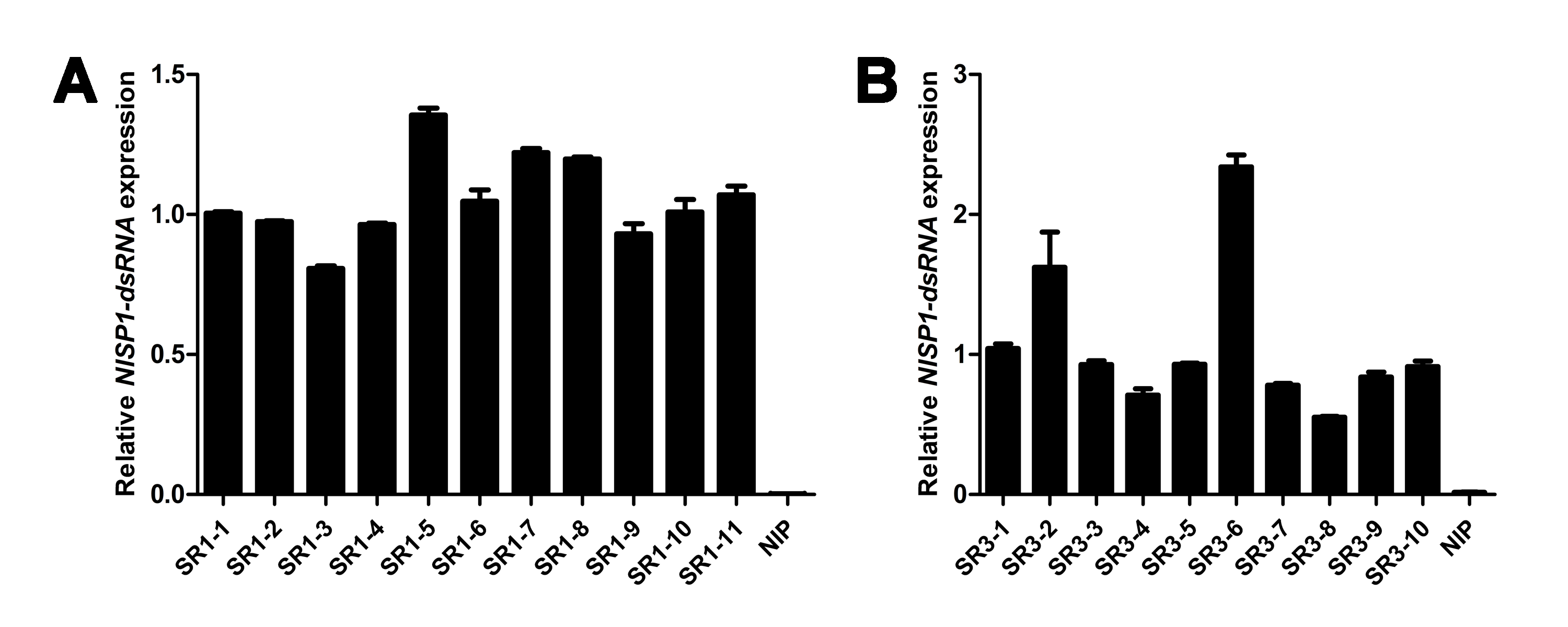

Supplement: Figure S6 — The relative expression of NlSP1-dsRNA in two independent NlSP1-RNAi T1 transgenic plants. (A) The relative expression of NlSP1-dsRNA in T1 transgenic plants of SR1. NIP, wild-type plant Nipponbare; SR1, an independent transgenic line expressing NlSP1-dsRNA. SR1-1–11, 11 T1 transgenic plants of SR1. (B) The relative expression of NlSP1-dsRNA in T1 transgenic plants of SR3. SR3, an independent transgenic line expressing NlSP1-dsRNA. SR3-1–10, 10 T1 transgenic plants of SR3. [file Image_6.tif]
